# Information Transmission and Processing in G-Protein-Coupled-Receptor Complexes

**Published:** 2025-08-14

**Authors:** Roger D. Jones, Achille Giacometti, Alan M. Jones

**Affiliations:** a Department of Biology University of North Carolina at Chapel Hill Chapel Hill North Carolina 27514 USA; b Dipartimento di Scienze Molecolari e Nanosistemi Universit“‘a Ca’ Foscari Venezia 30123 Venezia Italy; c European Centre for Living Technology (ECLT) Ca’ Bottacin 3911 Dorsoduro Calle Crosera 30123 Venezia Italy; d Department of Pharmacology University of North Carolina at Chapel Hill Chapel Hill North Carolina 27514 USA

**Keywords:** G Protein-Coupled Receptor (GPCR), information flow, entropy, Second Law of Thermodynamics, phosphatse, nonequilibrium steady state, induced fit

## Abstract

G-protein-coupled receptors (GPCRs) are central to cellular information processing, yet the physical principles governing their switching behavior remain incompletely understood. We present a first-principles theoretical framework, grounded in nonequilibrium thermodynamics, to describe GPCR switching as observed in light-controlled impedance assays. The model identifies two fundamental control parameters: (1) ATP/GTP-driven chemical flux through the receptor complex, and (2) the free-energy difference between phosphorylated and dephosphorylated switch states. Together, these parameters define the switch configuration. The model predicts that GPCRs can occupy one of three quasi-stable configurations, each corresponding to a local maximum in information transmission. Active states support chemical flux and exist in an “on” or “off” switch configuration, whereas inactive states lack flux, introducing a distinction absent in conventional phosphorylation models. The model takes two ligand-derived inputs: fixed structural features and inducible conformations (e.g. cis or trans). It shows that phosphatase activity, modeled as an energy barrier, chiefly governs “on”/“off” occupancy, whereas the kinase sustains flux without directly determining the switch configuration. Comparison with experimental data confirms the predicted existence of multiple quasi-stable states modulated by ligand conformation. Importantly, this framework generalizes beyond GPCRs to encompass a wider class of biological switching systems driven by nonequilibrium chemical flux.

## 1. Introduction

Biological systems act as computational networks of coupled chemical reactions that function as molecular switches. These networks transmit and process information to regulate cellular responses to environmental changes. Unlike neural computation, molecular information processing occurs primarily on membranes throughout the organism, with the immune system serving as a prominent example.

Malfunctioning molecular information networks often lead to disease, and most pharmaceutical approaches often aim to alter their function to improve health outcomes. With the advent of precision medicine [29, 31, 16], drug interventions must now be more fine-tuned, allowing more controlled and targeted modulation of these networks to produce optimal therapeutic effects.

A key example of molecular computation that we consider here is G protein-coupled receptors (GPCR), which regulate cellular responses to external stimuli and enable adaptation in diverse environments [41, 34]. Through interactions with downstream cytoplasmic couplers such as G proteins and β-arrestins, GPCRs control critical physiological processes [33, 12]. However, a single GPCR can drive both therapeutic and adverse outcomes, as illustrated by opioid receptors. G-protein coupling mediates analgesia, while β-arrestin coupling contributes to addiction [28, 30, 10]. This duality underscores the clinical challenge of modulating GPCR signaling to enhance benefit while limiting harm.

Like artificial computation, biological information processing requires sustained energy input and heat dissipation. GPCR switches operate far from thermodynamic equilibrium, driven by nucleotide hydrolysis (ATP/GTP) and phosphorylation/dephosphorylation cycles [3]. In the absence of this chemical flux, the systems would relax toward equilibrium, a state incompatible with life. From an evolutionary perspective and consistent with nonequilibrium thermodynamics and information theory, we propose that molecular computation is shaped by selective pressures favoring maximal information capacity and efficient energy use [39, 25, 54].

The mechanistic understanding of this molecular computation requires time-resolved assays capable of probing stimulus-response dynamics within the GPCR complex. Although prior work has mapped the input-output relationships of GPCR [9, 35]; Wirth et al. [55] advanced this effort by using photo-isomerizable ligands to modulate the GPCR conformation in real time and measure receptor activity via membrane impedance.

In this study, we extend previous theoretical work [23, 24, 22] to interpret these findings. Our model identifies two fundamental controllers of GPCR switching: (1) chemical flux through the phosphorylation cycle and (2) difference in free energy between phosphorylated and dephosphorylated configurations, both influenced by the chemically fixed and inducible conformations as well as extracellular ligand concentration.

This study demonstrates that GPCR switches operate in three distinct configurations, ”on,” ”off,” and inactive, contrasting with the conventional two-state view that conflates ”off” and inactive. Determining the switch state in this framework requires more than a single bit of information from the ligand. That information can be encoded in both the fixed chemical structure of the ligand and its inducible conformations. The fixed structures remain unchanged after receptor binding, while the inducible structures shift in response to external stimuli such as light or conformational changes arising from ligand–receptor interactions.

Experimental results confirm that Z (cis) and E (trans) ligand isomers modulate transitions between distinct “on”/“off” states and between active (flux-generating) and inactive (low-flux) modes. These discrete switching behaviors are directly related to the finite chemical flux that sustains the system.

Our model identifies two primary control inputs: chemical flux within the switch and the binding energy of γ-phosphates (or nucleotides in G-protein switches). By mapping switch conformations in this control space, the study offers a framework for the targeted modulation of biological switches, an essential step toward precise therapeutic control. A key unresolved question is how the ligand properties modulate these two control parameters.

## 2. Methods

### 2.1. Schematic of the Experiment

**Figure 1:**
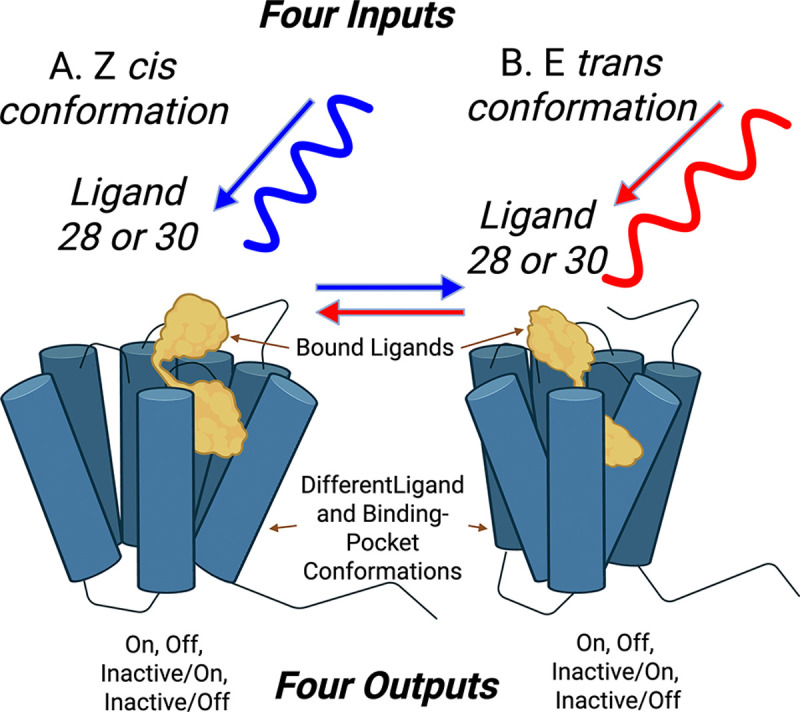
Conceptual schematic of the experiment. Ligands in cis (Z) or trans (E) configurations bind to GPCRs, while electrical impedance across a cell layer monitors signaling activity. Two engineered ligands (28 and 30) reversibly switch between Z and E isomers under wavelength-specific light (blue: Z→E; red: E→Z). Each conformation induces a distinct GPCR state, yielding four input combinations—encoding up to two bits of information. Experimental outputs map to four theoretical states: Active/On, Active/Off, Inactive/On, and Inactive/Off. However, impedance measurements cannot distinguish Inactive/On from Inactive/Off, so these are treated as a single ‘Inactive’ state. Theoretical modeling differentiates Active (flux-generating, dissipative) from Inactive (flux-free, non-dissipative) states

A conceptual schematic of the experimental setup from Wirth et al. [55] is shown in Figure 1. Chinese hamster ovary (CHO) cells were genetically engineered to overexpress the human pancreatic polypeptide receptor (hPP), a member of the neuropeptide Y family, and cultured to confluence on a gold foil substrate [5]. This uniform expression of the receptor enabled for consistent detection of ligand-induced conformational changes via electronic impedance measurements across the gold foil.

Two wavelength-driven ligand isomers, cis (Z) and trans (E), were derived from azobenzene (ligand 28) and arylazopyrazole (ligand 30), producing four distinct GPCR inputs: E-28, Z-28, E-30, and Z-30. Z isomers were generated by 340 nm irradiation, while E-isomers were produced using 455 nm (ligand 28) and 528 nm (ligand 30) light. GPCR response was inferred from changes in cell morphology, monitored by impedance measurements [48, 49, 53].

### 2.2. Schematic of the Theory

To regulate cellular responses to external stimuli, biological information-processing systems must operate persistently and far from equilibrium, placing them in non-equilibrium steady states (NESS), a dynamic form of homeostasis. These states are maintained by continuous energy flow from nucleotide hydrolysis (e.g., ATP, GTP), with entropy dissipated into the thermal bath.

The information content of an NESS remains stable over time, suggesting that it corresponds to a local or global extremum in information transmission. This aligns with the hypothesis that natural selection favors systems that maximize information flow under physical constraints. To formalize this, we quantify the information using mutual information [47] and apply the calculus of variations to identify the extrema, constrained by the constant chemical flux supplied by the input of nucleotide-driven energy.

## 3. Results

### 3.1. Experimental results relevant to this study

If the four inputs produce four independent outputs, this corresponds to two bits (${log}_{2}4$) of information transmitted across the membrane. Fewer outputs imply less information transfer. If only the chemical structure of the ligand (for example, 28 vs. 30) affects the response, only one bit is fixed. Observing more than two distinct responses implies that both fixed- and induced-fit [13] ligand conformations contribute to signaling.

The experiment in [55] showed four distinct responses, indicating that both the Z / E conformation and the fixed conformation of the ligand are very important. This suggests that each ligand exists in two functionally distinct conformational states after light activation, resulting in the full utilization of two bits of information in the signaling process. These findings align with independent observations of GTPase activity in other receptors showing similar two-bit transmission [27].

The ligand conformation was found to be reversibly switched between *cis* and *trans* even while the ligand remains bound to the receptor. Notably, removing ligands from the extracellular fluid does not alter the downstream response, indicating that signaling is determined by the conformation of the bound ligand rather than its continued presence in solution.

### 3.2. Theoretical Results

The model described in Sections Appendix A.1, Appendix A.2, and the Supplement shows that a molecular switch can occupy quasistable states determined by two parameters: the chemical flux within the GPCR complex and the switch’s free energy change. Both parameters are influenced by the extracellular ligand’s characteristics—its fixed and inducible conformations as well as its concentration outside the cell.

This framework leads to the Biological Ensemble, a generalization of the Canonical Ensemble from equilibrium statistical mechanics [42]. The Canonical Ensemble, described by Feynman as the “summit of statistical mechanics”^1^ [11, Chapter 1], admits a single solution proportional to the exponential of the system energy [42]. In contrast, the Biological Ensemble introduced here yields three possible solutions that turns the ensemble into a system of switches, a key result of this work.

We illustrate the power of the Biological Ensemble with an intuitive analogy. Near thermochemical equilibrium, a system relaxes to a single state, much as air molecules uniformly fill a room at fixed temperature and density. In our framework, if those molecules were in an NESS, the room could instead exist in one of three distinct states: one resembling the usual equilibrium condition and two others in which all molecules cluster into two different corners of the room. In this analogy, the NESS behaves as a switch with three possible configurations. It is clear that a room in equilibrium and a room in NESS are fundamentally different, and each would exhibit markedly different behavior.

The solutions correspond to local maxima of information flow, quasistable states that can transition under perturbation. Two parameters govern which state the switch occupies: (1) the energies of the phosphorylated ($E_{p}$) and dephosphorylated ($E_{d}$) conformations, and (2) the chemical flux $J_{0}$ from the phosphorylation/dephosphorylation cycle. Together, these parameters determine whether the switch is in the “off”, “on” or inactive configuration.

The energy parameter is given by the normalized change in the Gibbs free energy in going from the “off” to “on” switch configuration.

(1) $$\beta \left(E_{p}-E_{d}\right)$$

where β is the inverse of the temperature of the heat bath (Boltzmann constant = 1). The Gibbs free energy is the valid form of free energy to use since the system is in a heat bath at temperature T=1/β and no external work is being applied to the system. The energies $E_{p}$ and $E_{d}$ are modified from their baseline measured values (≈ 7 kcal/mol) [43] by the presence of the protein matrix of the GPCR complex. Modifications as high as 17 kcal/mol have been seen in the muscles of athletes [52].

If $R_{p}$ and $R_{d}$ are the numbers of receptors in the phosphorylated (“on”) and dephosphorylated (“off”) switch configurations, then the probability of the receptor being in each of the switch configurations is

(2) $$Pr(p)=\frac{R_{p}}{R_{d}\;+\;R_{p}}$$

and

(3) $$Pr(d)=1-Pr(p)=\frac{R_{d}}{R_{d}\;+\;R_{p}}$$

for the phosphorylated and dephosphorylated configurations, respectively.

We define the chemical flux $J_{0}$ as the flow of probability from one switch configuration to the other.

(4) $$J_{0}=Pr(d)\;Pr(p|d)=Pr(p)\;Pr(d|p)$$

where Pr(p|d) is the probability that the receptor state transitions from configuration d to p and Pr(d|p) is the probability of transition in the opposite direction. The equality sign is a consequence of the steady-state requirement and is an expression of Bayes Theorem [17].

The terms in Equation 4 are intuitive from a chemist’s perspective. The probability Pr(d) is proportional to the chemical concentration of the system in the dephosphorylated configuration, while Pr(p) is proportional to the concentration of the phosphorylated concentration. The transition probabilities Pr(p|d) and Pr(d|p) are the reaction rates.

An intuitive schematic of the information landscape is provided in Appendix C, illustrating how the system navigates between quasistable states. The figure represents a landscape in which the curves are ridges of maximum switch information. Three solutions exist for the probability Pr(p) that the switch is in the “on” configuration for each flux and change in free energy value. In the limit of small flux, the solutions are (1) Pr(p)=1, Pr(d)=1-Pr(p)=0 (blue), (2) Pr(d)=1-Pr(p)=1, Pr(p)=0 (red), and (3) the Canonical Ensemble (yellow). We define solution (3) as the thermodynamic branch, while solutions (1) and (2) are defined to be the kinetic branch.

For small values of flux $J_{0}$, the highest information path, the thermodynamic branch (yellow), for probability Pr(p) obeys the Canonical Ensemble. The thermodynamic branch steepens as the chemical flux $J_{0}$ increases. At $J_{0}=\phi_{c}\approx 0.182$, the information content of the kinetic branch becomes equal to the information of the thermodynamic branch. For $J_{c}>\phi_{c}\approx 0.182$, the information in the kinetic branch is greater than the information in the Thermodynamic Branch. This implies that for flux greater than $\phi_{c}$, the states in the kinetic b ranch are more stable than the states in the thermodynamic branch. The opposite is true for $J_{0}<\phi_{c}$.

At $J_{0}=1/4$, the thermodynamic branch is given by Pr(p)=0. For $J_{0}>1/4$, a discontinuity forms in the kinetic branch and the two solutions exchange roles as the change in free energy increases.

**Figure 2:**
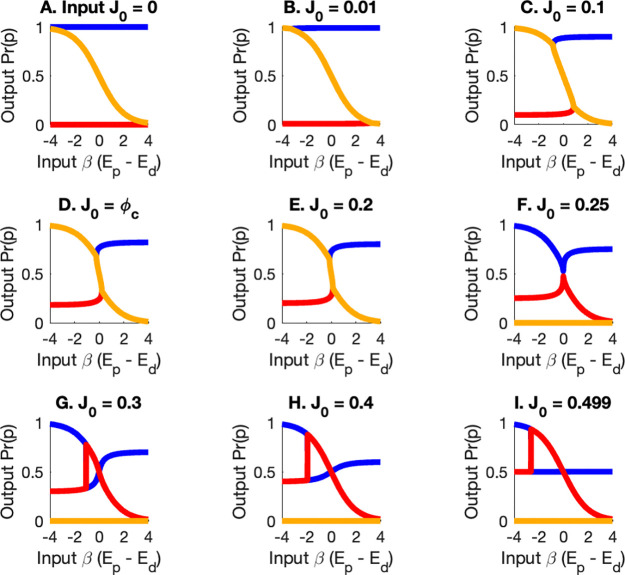
The three solutions for Pr(p) for the Biological Ensemble as a function of the chemical flux $J_{0}$ and change in free energy between two receptor states $\beta \left(E_{p}-E_{d}\right)$. **A.** In the limit of small flux, the solutions are (1) Pr(p)=1, Pr(d)=1-Pr(p)=0 (blue), (2) Pr(d)=(1-Pr(p)=1, Pr(p)=0 (red), and (3) the Canonical Ensemble of (yellow). **B.-F.** For small values of flux $J_{0}$, The highest information path for probability Pr(p) obeys the Canonical Ensemble. The thermodynamic branch steepens as the chemical flux $J_{0}$ increases. **G.-I.** A discontinuity forms in the kinetic branch and the two solutions exchange roles with increasing change in free energy. For fluxes less that the critical flux $\phi_{c}\approx 0.182$

The curves in Figure 2 represent local maxima of information transmission, corresponding to quasi-stable states defined by chemical flux $J_{0}$ and change in free energy $\beta \left(E_{p}-E_{d}\right)$. Here β is the inverse temperature of the heat bath, $E_{p}$ is the energy of the phosphorylated “on” state and $E_{d}$ is the energy of the dephosphorylated “off” state. These configurations act as local attractors, indicating preferred switch states within the control space.

Observed switch behavior is expected to align with these quasi-stable states. The Biological Ensemble framework shows how variations in $J_{0}$ and $\beta \left(E_{p}-E_{d}\right)$ guide the system into specific configurations, offering predictive insight into the switch dynamics under varying conditions.

A simple water-pump analogy, presented in the Discussion, offers a macroscopic visualization of the mechanism by which the switch transitions between these states.

### 3.3. Comparison of Theory and Experimental Results

**Figure 3:**
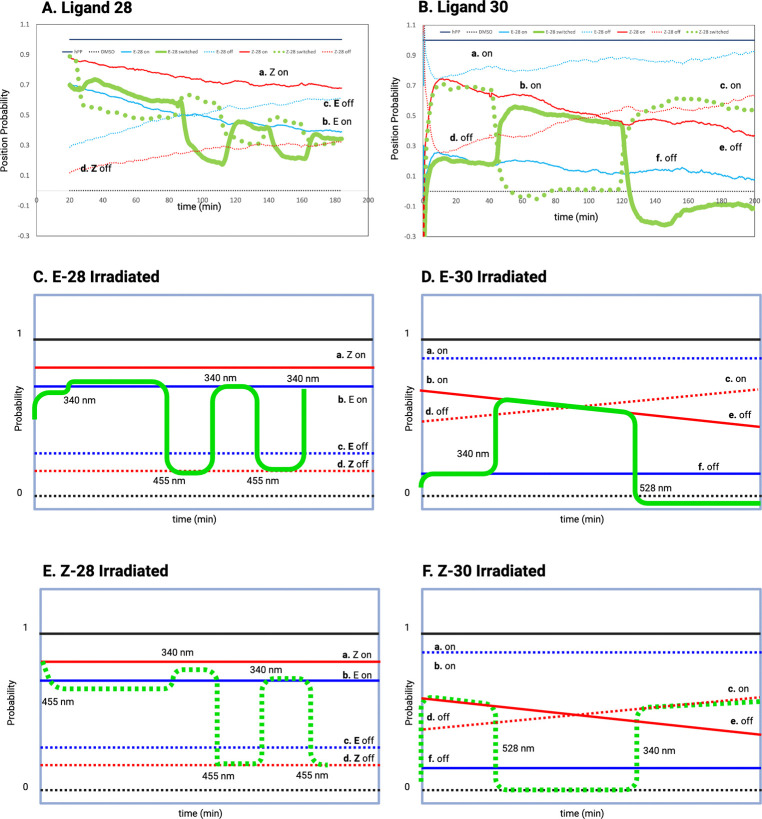
**A. and B.** Experimental Results **C.-F.** Schematic of Quasistable States. For Ligand 28, irradiation with wavelengths 340nm and 455 nm toggle the the system between the E/on (trans) and the Z/off (cis) states. For Ligand 30, irradiation with 340 nm and 528 nm toggles between active and inactive configurations.

The experimental results from Wirth et al. are depicted in Figures 3A and B, alongside the theoretical predictions. The mapping from impedance to probability is given in Appendix B. Solid and dashed red lines show impedance responses for ligands 28 and 30 initially prepared in the Z/on and Z/off configurations, respectively; blue lines represent E/on (solid) and E/off (dashed) preparations. The solid black line shows the response to the endogenous ligand hPP, and the dashed black line corresponds to the solvent control (DMSO). Figure 3A shows the results for ligand 28, and Figure 3B for ligand 30. Green lines indicate ligands prepared in the Z/on (dashed) and E/on (solid) states that were alternately irradiated with short (340 nm) and long (455 nm for 28; 528 nm for 30) wavelengths.

Figure 3C–F present schematic summaries of the experimental transitions among quasi-stable switch configurations. Figure 3C shows that for ligand 28 in the E/on configuration, the impedance remains similar to that of Z/on. The off” states (E/off and Z/off) also overlap. Initial 340 nm irradiation slightly increases impedance but does not shift the system out of E/on. Subsequent alternating irradiation causes the system to toggle between E/on and Z/off, indicating that cis (Z) favors the “on” state and trans (E) favors “off.”

Figure 3E shows the system initialized in the Z/on state. After a brief stable period, alternating light induces switching between Z/off and E/on, again confirming the correlation between the cis/trans states and switching on/off for ligand 28.

For ligand 30 (Figures 3D and F), the Z/on and Z/off responses overlap, indicating that the “on”/“off” distinction does not apply. Instead, the system transitions between an active Z state that supports chemical flux and an inactive E state that does not. Thus, ligand 30 modulates switch activity versus inactivity, rather than discrete “on”/“off” signaling.

In summary, ligand 28 toggles the switch between on and off based on Z/E conformation, while ligand 30 toggles between active and inactive states. These behaviors reflect different modes of ligand control over GPCR switching.

## 4. Discussion

Biological systems operate far from thermochemical equilibrium and depend vitally on continuous entropy flow sustained by external chemical fluxes, denoted as $J_{0}$. This flux represents the transition of GPCR receptor states among inactive, “off,” and “on” configurations. In advanced biological information-processing units such as GPCR complexes, the erasure of information unavoidably generates heat and entropy [32], emphasizing the thermodynamic requirement for chemical flux; in its absence, biological computation cannot occur.

To capture the behavior of these nonequilibrium steady states (NESS), we introduce the Biological Ensemble, a theoretical framework that extends classical free-energy minimization by explicitly incorporating both chemical flux and information flow. This formulation, developed in detail in Appendix A.1, enables the prediction of switch dynamics that is beyond the reach of traditional equilibrium models.

Our model reveals two distinct behavioral regimes. Near equilibrium, with small $J_{0}$, the systems operate within the thermodynamic branch, where physics is dominated by thermal effects. However, as $J_{0}$ increases, driving the system further from equilibrium, behavior transitions to the kinetic branch, where non-linear dynamics dominate.

The thermochemical equilibrium solution, corresponding to the thermodynamic branch, is shown as the yellow curve in Figure 2A. This solution represents a global maximum of information flow through the system. In contrast, the red and blue curves represent additional non-equilibrium steady states (NESS) with lower overall information content. These states are nevertheless local maxima of information flow and are therefore quasistable. Although they are absent in an equilibrium treatment of the switch, they emerge naturally in the nonequilibrium framework and represent valid, physically realizable configurations.

As the chemical flux $J_{0}$ increases, the landscape of quasistable solutions diverges markedly from the prediction of thermochemical equilibrium. At a critical flux value $\phi_{c}$, the red and blue solutions of the kinetic branch surpass the thermodynamic (yellow) branch in information content. Beyond this point, the solution that initially corresponded to the thermochemical equilibrium state transitions to a strictly “off” configuration, as illustrated in Figures 2F–I.

The control parameters, $\beta \left(E_{p}-E_{d}\right)$, representing the difference in free energy between phosphorylated and dephosphorylated states, and $J_{0}$, the chemical flux, are themselves subject to upstream regulation. Ligand-induced conformational changes in the GPCR complex can modulate the free energy landscape, effectively tuning $\beta \left(E_{p}-E_{d}\right)$. Meanwhile, flux $J_{0}$ depends on the concentration and binding of extracellular ligands; In the absence of a bound ligand, flux cannot be sustained. Thus, fixed and inducible conformations of the ligand and availability serve as higher-order controls over switch behavior.

One must be cautious in using common biochemical analysis techniques, such as determining the structure of the system by minimizing the free energy, that do not apply to information processing systems that require continuous energy and entropy input.

To transition the switch among inactive, “off,” and “on” configurations, the system must transition among quasistable states. Such transitions correspond to the movement of the system through the information landscape illustrated in Figures 2 and C.6. The two inactive states are indistinguishable from each other. These shifts can be induced by allosteric conformational changes in the receptor complex triggered by binding of the extracellular ligand. This study does not address how this allosteric interaction occurs.

We applied this framework to analyze the signaling of GPCRs, specifically how two bits of ligand input, chemically fixed (28 or 30) and inducible conformations (Z or E), translate into four distinct GPCR output states: active/on, active/off, inactive/“on” and inactive/“off”. Our model distinguishes active states by the presence of flux and heat dissipation, contrasting them with inactive states, where flux is small. The ”on/off” distinction is attributed to phosphorylation or nucleotide binding. This full two-bit information transmission is consistent with prior measurements from one-switch systems. The observation of four distinct outputs, under the assumption of input independence, is particularly significant. Had inputs like Z-28 and E-28 been correlated, fewer outputs would result. This suggests that photoisomerization induces conformational changes that are more intricate than simple Z/E geometry, emphasizing the critical role of ligand preparation beyond a mere chemically fixed conformation. In addition to chemically fixed and inducible conformations, the concentration of extracellular ligands also carries information [22].

For simplicity and clarity, we selected the phosphorylation / dephosphorylation cycle as the representative switch. However, the theoretical framework is broadly applicable to any binary chemical reaction-based switch. A particularly relevant example, especially in the context of GPCR signaling, is the nucleotide exchange reaction, which shares the core functional elements of phosphorylation-based switches [41]. Given the nature of GPCR coupling, it is likely that the switching observed in the Wirth et al. experiment reflects nucleotide-driven mechanisms.

Biological switches function within a complex matrix of dynamic partner interactions, inducible conformations shaped by the solvent environment (e.g., the membrane), and covalent modifications such as lipidation and disulfide bridging. These factors influence both the free energy and the chemical flux of the switch. A strength of the thermodynamic approach is that many predictions can be made without knowing these details. For example, a ball in a bowl will settle at the bottom—the lowest energy state—regardless of the exact forces involved or its initial position; all that matters is that friction exists and the bowl has a minimum. Likewise, for molecular switches, if the goal is to identify their final NESS, the precise trajectory to that state is often irrelevant.

**Figure 4:**
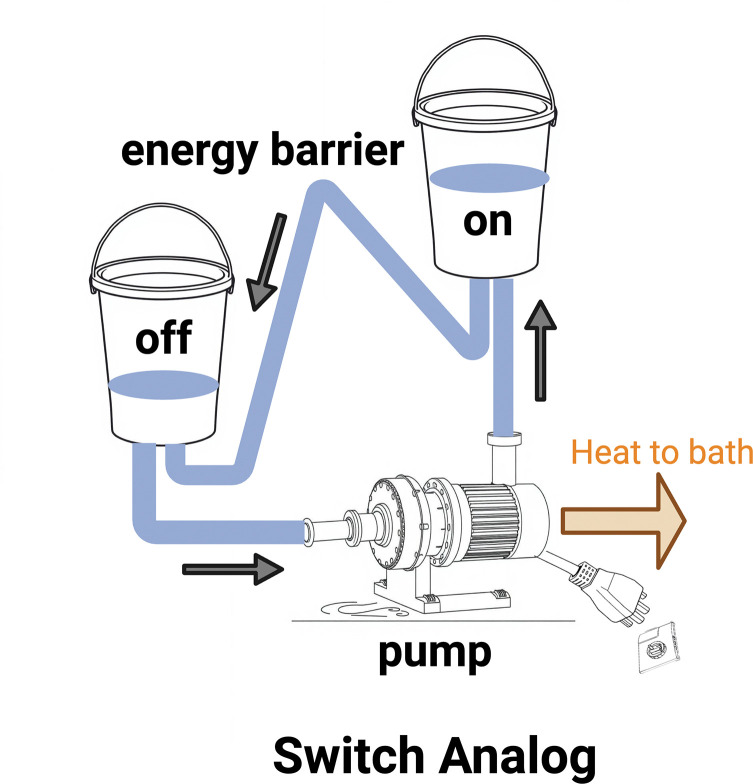
The “on” and “off” switch conformations are modeled as water buckets labeled on and off, where water levels represent the probability of each receptor state. Forward flow fills the on bucket, while reverse flow fills the off bucket. A pump, analogous to ATP/GTP-driven energy input, powers the cycle. The flow magnitude and state occupancy are regulated by an energy barrier, represented by the height of the return tube, which controls resistance to reverse flow.

A simple and intuitive analog for the switch mechanism using water pumped between two buckets is shown in Figure 4. A water molecule in bucket **off** represents the probability that the switch is in the “off” state, while a molecule in bucket **on** represents the “on” state. The water pump symbolizes a kinase, which drives the transition from **off** to **on** using energy supplied by ATP or GTP. Relative probabilities (bucket water levels) are modulated by an energy barrier, represented as the height of the hose, representing the activity of a phosphatase. The heat generated by the pump and the water flow is dissipated into the surrounding heat bath.

The figure shows that the phosphatase, represented by the energy barrier, primarily controls the relative occupancy of the “off” and “on” switch configurations. Increasing the barrier height shifts occupancy toward the “on” state, while lowering it favors the “off” state. If the barrier is raised too high, the switch locks in the “on” state and the pump does not have a water substrate to maintain flux. In contrast, the kinase, illustrated as the pump, mainly serves to maintain the chemical flux through the cycle and does not directly influence the occupancy.

Although the present study does not address the phosphorylation barcode, which is known to influence β -arrestin signaling, future work will investigate how ligand concentration might encode additional information through this mechanism. A central goal will be to determine the sequence in which switches are activated and deactivated as the extracellular ligand concentration varies. This question can be experimentally addressed using response curves, and preliminary theoretical considerations have been outlined in our earlier work [22].

The theory developed in this study was applied specifically to GPCR-mediated information processing due to the ubiquity and therapeutic significance of GPCRs, which are targets for approximately 30% of all drugs [44]. However, the theoretical framework is broadly applicable and not limited to GPCR complexes. Phosphorylation/dephosphorylation cycles function as ubiquitous molecular switches in biological systems, translating transient or graded inputs into discrete, regulated outputs. These cycles are fundamental to cellular decision-making and underlie numerous signaling pathways, including Receptor Tyrosine Kinases (RTKs) [46] and Toll-like Receptors (TLRs) [6]. Moreover, as shown in this study, biological switches are not limited to phosphorylation/dephosphorylation cycles; nucleotide exchange-driven switches are equally relevant in GPCR signaling. The generality of the theory makes it relevant to a wide range of biological switching systems beyond those explored here.

## 5. Conclusions

1. A first-principles theoretical model was developed to explain the GPCR switching behavior observed in published dynamical GPCR-activation data [55].
2. The model identifies two key drivers of the switching: chemical flux within the GPCR complex and the free-energy difference between the phosphorylated and dephosphorylated switches.
3. For a given flux, the switch can occupy one of three quasi-stable states, each representing a local maximum in information transmission (Figure 2). Active switches support flux and can exist in either ”on” or ”off” states; inactive switches do not support flux.
4. The quasistable states can be modulated *in situ* by changing the ligand conformation between *cis* and *trans* forms.
5. The phosphatase plays the central role in determining “on” and “off” configurations of the switch.

## Figures and Tables

**Figure A.5:**
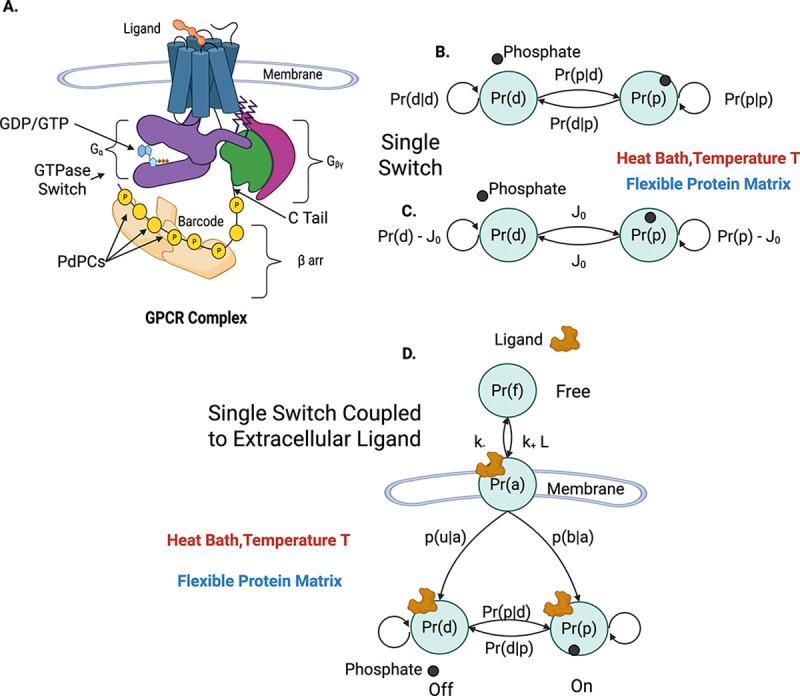
Simple Molecular Switch A. The 7-transmembrane GPCR is illustrated with blue cylinders representing the seven α helices that span the cell membrane. The extracellular ligand (orange) binds to the binding site of the GPCR inducing movement with the α helical bundles. The helices alter the conformation of the intracellular loops (thin black lines) of the GPCR complex. The intracellular portion of the complex has been separated for visibility. Two pathways may be activated, the $G_{\alpha }$ pathway (purple) and the βarr pathway (tan). The $G_{\alpha }$ subunit is a part of the G protein also composed of subunits β and γ. The βarr pathway is composed of additional response pathways determined by phosphorylation sites on the C tail of the GPCR and intracellular loops that form a barcode that encodes signals for downstream processes. **B.** Picture of a single switch. The switch resides in a heat bath at temperature T. In addition to the thermal bath, the switch is a component of a flexible protein complex that can modify energy barriers in the switch. Here, Pr(d) and Pr(p) are the probabilities of finding the receptor to be unbound and bound to a ligand, respectively, while Pr(p|d) and Pr(d|p) are the conditional probabilities of finding the receptor has transitioned between phosphorylated to dephosphorylated positions and *vice versa*. **C.** Here, $J_{0}\;=\;Pr(p|d)\;Pr(d)\;=\;Pr(d|p)\;Pr(p)$ is the steady-state probability flux among states. The flux is kept finite due to an external energy/entropy source. **D.** Three-state model: free receptor (f), phosphorylated (p), and dephosphorylated (d). The associated state a is bound to the ligand but not the phosphate. The associated state a has two internal states d and p. These two states form the $G_{\alpha }$ switch. Ligand dissociation and association reaction rates are given by $k_{-}$ and $k_{+}$, respectively, and L is the ligand concentration.

**Figure C.6:**
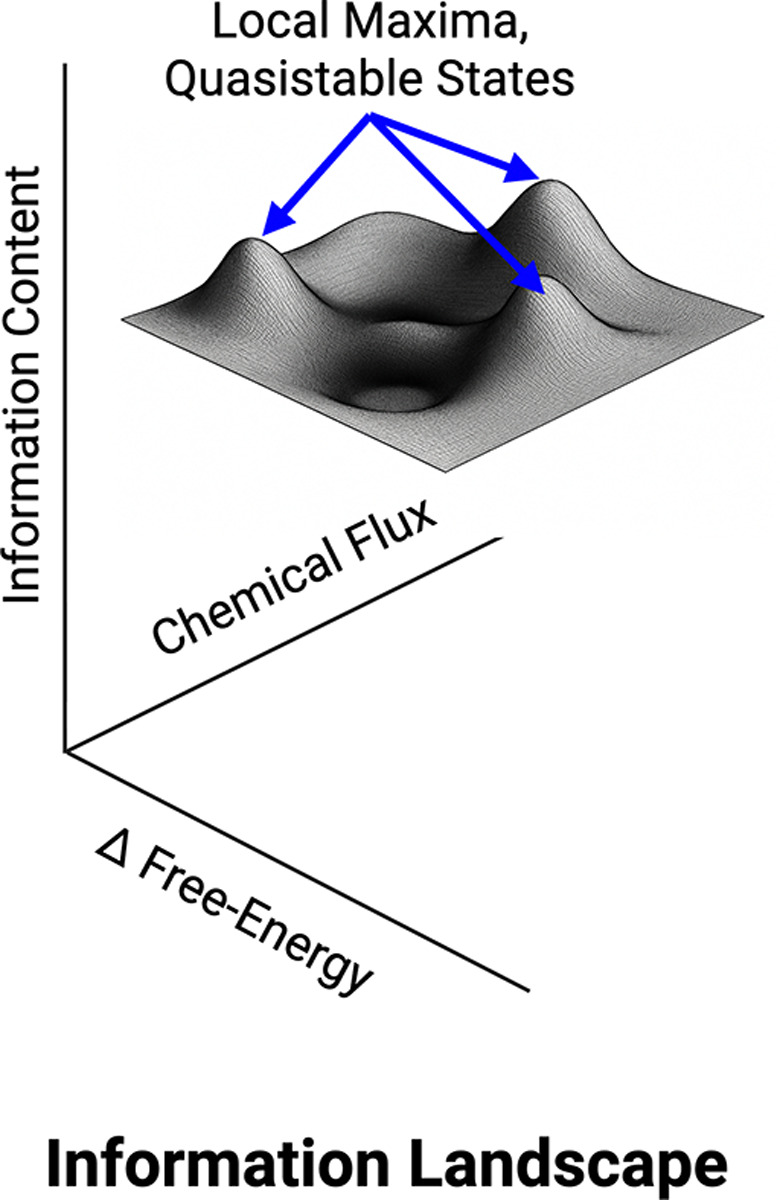
The two control parameters are given by the change in the free energy of phosphate bonding, $\beta \left(E_{p}-E_{d}\right)$, and the chemical flux, $J_{0}$. The information content of the system is displayed on the vertical axis. Local maxima and minima of the information are locations of steady state for information content of the system.

**Figure C.7:**
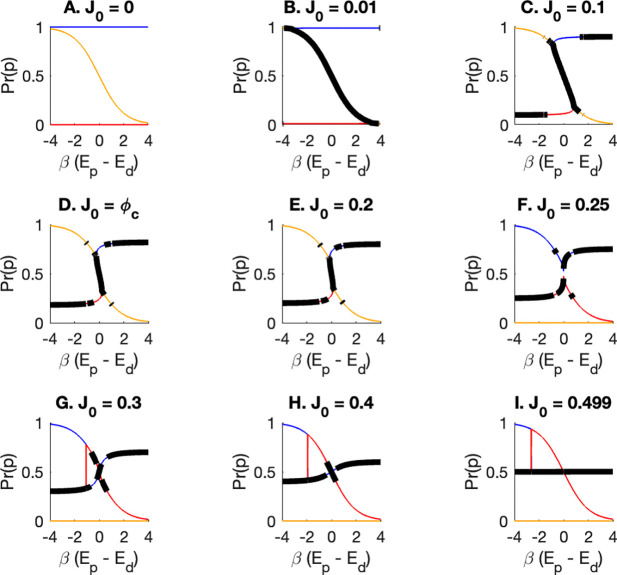
The paths with the largest information transmission are outlined in black. **A.** In the limit of zero flux, all paths have the same information transmission. **B.** For a very small but finite flux $J_{0}$, the thermodynamic branch has the highest capacity. The highest capacity path for probability Pr(p) obeys the Canonical Ensemble. **B.-I.** For a given flux $J_{0}$, discontinuities in the high capacity path appear with changes in binding energy $\beta \left(E_{p}-E_{d}\right)$ The highest-capacity path represents a global maximum, while the other possible paths represent local maximum, or quasi-stable states of the switch.

## Highlights

- A first-principles theory of molecular switches, validated by experiment, is presented.
- The model predicts three switch configurations: on, off, and inactive.
- Switch state is controlled by free energy difference and externally driven chemical flux.
- Targeting dephosphorylation rates offers a potential drug strategy for switch modulation.

## Footnotes

1 “This fundamental law is the summit of statistical mechanics, and the entire subject is either the slide-down from this summit, as the principle is applied to various cases, or the climb-up to where the fundamental law is derived and the concepts of thermal equilibrium and temperature T clarified.” R. P. Feynman

## Appendix A. Theory

### Appendix A.1. Information and the Second Law of Thermodynamics

Standard techniques that analyze systems by minimizing free energy do not apply to information-processing systems. Because information processing requires continuous energy and entropy flow, even in steady state [32], entropy maximization is not achievable in non-isolated systems.

The entropy S of a system composed of W states $x_{i}$ is

(A.1)
$$S=-\sum_{i=1}^{W}Pr\left(x_{i}\right)ln\;Pr\left(x_{i}\right)$$

Assume that the system is composed of a switch in the form of a chemical reaction

(A.2)
u⇌b

where, as an example, d is the dephosphorylated state of the switch and p is the phosphorylated state of the switch. The rest of the system is a heat bath that absorbs heat that is generated by the chemical reaction. The entropy, Eq. A.1, can then be written

(A.3)
$$S=S_{B}-I_{S}$$

where $S_{B}$ is the entropy of the heat bath given that the observables d and p is the switch are independent and $I_{S}$ is the information in the correlation of the components d and p of the switch. The heat bath entropy $S_{B}=S_{S}+S_{x}$ is composed of two pieces, the entropy $S_{S}$ of uncorrelated components of the switch and the entropy $S_{x}$ of the microstates x. The entropy $S_{S}$ of the uncorrelated switch components is

(A.4)
$$S_{S}=-Pr(d)ln\;Pr(d)-Pr(p)ln\;Pr(p)$$

while the entropy of the microstates is

(A.5)
$$S_{x}=-\sum_{i=1}^{W}Pr\left(x_{i}\right)ln\left[\frac{Pr\left(x_{i}\right)}{Pr(d,p)}\right]$$

where Pr(d,p) is the joint probability of d and p.

The mutual information [47] associated with the correlation of d and p is

(A.6)
$$I_{S}=\sum_{\left\{d,p\right\}}Pr(d,p)ln\left[\frac{Pr(d,p)}{Pr(d)Pr(p)}\right]$$

The Second Law of Thermodynamics states that the entropy of the system increases with time or achieves a maximum value.

(A.7)
$$\Delta S=\Delta S_{B}-\Delta I_{S}\geq 0$$

If the number of states W is infinite, the system never achieves a maximum value.

In the case that the switch achieves a steady state $\Delta I_{S}\to 0$, then all the entropy generated by the switch goes into the heat bath.

(A.8)
$$\Delta S\to \Delta S_{B}\geq 0$$

When the switch has reached steady state $\Delta I_{S}\to 0$, the switch information $I_{S}$ is at a maximum or a minimum.

For a system with an infinite number of states W, total entropy continues to increase indefinitely. Local regions of correlation, such as molecular switches, can achieve steady states even as the entropy of the larger system grows. The heat generated by the switch is transferred to the surrounding heat bath. This scenario contrasts with finite systems W, where the total entropy S can reach a maximum. Because biological information processing systems function far from equilibrium, traditional frameworks based on minimizing Gibbs free energy G=E-TS, with E as the energy of the system and T the temperature of the bath do not apply.

### Appendix A.2. Theoretical Model of the Switch

Building on our earlier theoretical framework [23, 24, 22], which successfully predicted the results of the experimental assay by maximizing the flow of information from the extracellular environment to the intracellular region of the GPCR complex, the present work extends this theory by incorporating a higher temporal resolution. This enhanced model is specifically developed to interpret the detailed, time-dependent dynamics observed in recent impedance-based experiments by Wirth et al. [55]. In line with its predecessor, this updated model is guided by the fundamental principle that natural selection favors molecular systems optimized to maximize information transmission capacity [47], thus providing a more granular and dynamic view of the GPCR switching mechanisms.

The foundation of the current model is illustrated in Figure A.5, which schematizes the GPCR complex. In Figure A.5A, the 7-transmembrane GPCR (blue) spans the membrane, interacting with both extracellular ligands (orange) and intracellular G proteins (purple, green, violet).

Ligand binding induces a conformational change in the GPCR, which enhances its affinity for heterotrimeric G proteins. This results in the formation of a ternary complex - comprising the ligand, GPCR, and G protein - that is more stable than the ligand receptor complex alone.[36].

The G protein α subunit ($G_{\alpha }$) initially binds to GDP, representing the off state of the GTPase switch [41]. Upon GPCR activation, GDP is released and replaced by GTP, whose cellular concentration is maintained far from equilibrium. This GTP binding activates $G_{\alpha }$, which completes the switch to the ”on” state. The energy driving this transition derives from the thermodynamic disequilibrium between GDP and GTP.

The C-terminal tail of GPCR and the intracellular loops contain serine and threonine amino acid sites that can be phosphorylated by specific kinases. The phosphorylation and dephosphorylation of these sites is known as a phosphorylation/dephosphorylation cycle (PdPC) that also acts as a molecular switch [41]. Similarly to the GTPase switch, the “on” and “off” states of these PdPC switches are determined by the phosphorylation status of specific sites. However, in this case, the switches are powered by the high concentration of adenosine triphosphate (ATP), which drives the process [2].

The collection of these PdPCs establishes a distinct molecular pattern, called a barcode, which serves as a guide for downstream signaling processes [56, 7]. Adapters read this phosphorylation barcode to propagate a certain response (Figure A.5). In animals, this adapter is called β-arrestin [33] and in plants it is called VPS26A/B [21].

Furthermore, the flexible protein matrix surrounding the GPCR complex provides a mechanism to adjust energy levels and transition probabilities, facilitating changes in the conformation and behavior of the complex.

Figures A.5B and A.5C presents a simplified mathematical representation of the schematic shown in Figure A.5A. This abstraction allows for a more tractable analysis using the tools of statistical mechanics [42]. This level of simplification is common and has been useful in physics [8, 19]. In this model, the GPCR complex is reduced to a single switch embedded in a heat bath and surrounded by a flexible protein matrix [26, 15, 37, 20, 45, 4, 51, 50, 18]. The probabilities Pr(d) and Pr(p) represent the fraction of time the receptor spends in the dephosphorylated and phosphorylated states, respectively. Specifically, the d position corresponds to the state in which the G protein is bound to a GDP molecule, while the p position corresponds to the state in which GDP has been phosphorylated to form GTP. During a time interval ∆t, the switch can transition between these states with transition probabilities Pr(d|p) and Pr(p|d), or it can remain in the same state with probabilities Pr(u|u) and Pr(b|b). The heat bath ensures that the fluctuations between these switching states occur as if the system is at a temperature T=1/β, where T is in energy units.

Figure A.5C describes the switch in terms of chemical flux

(A.9)
$$J_{0}=Pr(p|d)\;Pr(d)=Pr(d|p)\;Pr(p)$$

which represents the flow of probability from one switch position to another. This description is useful if the switch is fueled by an external source such as a GTP/GDP disequilibrium. A flow of energy from GTP/GDP into the switch is turned into heat $Q_{⊙}$ in the time interval ∆t

(A.10)
$$Q_{⊙}=J_{0}q=-\Delta G_{⊙}$$

where -q is the amount of heat released when a GPCR transitions from the “off” position to the “on” position and back; and ∆G is the amount of the free energy from GTP that is injected into the switch in time ∆t. The minus sign indicates that the heat is leaving the switch. Equation A.10 describes the flow of entropy through the switch. A flow of entropy of this nature is essential for life [38]. We take flux $J_{0}$ as the external driver of emergent behavior in the switching system.

The details of ligand/receptor binding are an important part of the overall picture. The inclusion of ligand kinetics is shown in Figure A.5D. The interaction between the ligand and the free receptor f to form the bound associated state a is modeled using Michaelis-Menten kinetics [3]. The forward reaction rate is given by $k_{+}L$ where L represents the ligand concentration, while the reverse reaction rate is $k_{-}$

Within the associated state a, the receptor can adopt different internal states corresponding to the switch positions. The probability that the receptor is in the dephosphorylated “off-”state d given that it is in a is denoted as Pr(u|a). Similarly, the probability that the receptor is in the phosphorylated “on” state is Pr(b|a). The observed probabilities Pr(d) and Pr(p) are

(A.11)
Pr(d)=Pr(a)Pr(u|a)

and

(A.12)
Pr(p)=Pr(a)Pr(b|a)

Aspects of the ligand/receptor binding kinetics were observed in [55]. Specifically, it was observed that the ligands remained bound to within the time range of the experiment even if the extracellular fluid was washed free of ligands. The details of these observations provide insight into the role that the switch position plays in ligand binding. In this study, we do not address this process. This will be addressed in a follow-up study.

The transmission of information I [47, 1, 14] through the switch is:

(A.13)
$$I=\sum_{i\in \{d,p\}}\sum_{j\in \{d,p\}}Pr(j|i)\;Pr(i)\;log\left(\frac{Pr(i|j)}{Pr(i)}\right)$$

(A.14)
$$I=J_{0}log\left(\frac{Pr(d|p)\;Pr(p|d)}{Pr(d)\;Pr(p)}\right)+\left(Pr(d)-J_{0}\right)log\left(\frac{Pr(u|u)}{Pr(d)}\right)+\left(Pr(p)-J_{0}\right)log\left(\frac{Pr(b|b)}{Pr(p)}\right)$$

Maximization of the information transmitted through the switch subject to the constraints that the total energy $\overset{‾}{E}$ of the switch is given and the flux $J_{0}$ is an externally determined constant leads to what we refer to as the Biological Ensemble

(A.15)
$$Pr(d{)}^{2}\left[Pr(p)-J_{0}\right]\;exp\left(-\beta E_{p}\right)=Pr(p{)}^{2}\left[Pr(d)-J_{0}\right]\;exp\left(-\beta E_{d}\right)$$

(A.16)
$$\overset{‾}{E}=Pr(d)\;E_{d}+Pr(p)\;E_{p}$$

where $E_{d}$ is the energy of the switch in the off position and $E_{p}$ is the energy of the switch in the on position. The word “ensemble” is commonly used in two related concepts. It can be used to describe the probability given by Eq. A.15 of finding a switch in the p position or it can describe an imaginary collection of switch states that have probability given by Eq. A.15 or another equation such as the Canonical Ensemble [42].

The Biological Ensemble, Eq. A.15, determines the theoretical outcomes and testable predictions that can be used to inform the experimental observations of [55]. Like the Canonical Ensemble in equilibrium thermodynamics [42], the Biological Ensemble can predict microscopically observable behavior such as the impedance measurements described in the previous section. To see this, we assumed that the switch positions are correlated with the GPCR conformations generated by the Z and E forms of the ligand. It follows that Eq. A.15 predicts the relationships among the impedance observations in [55]

Three solutions of the biological ensemble exist for small chemical flux $J_{0}=0$ (Figure 2A). One solution (yellow) corresponds to the Canonical Ensemble [42] valid for systems in thermal equilibrium. We refer to this solution for all permissible flux values $J_{0}$ as the Thermodynamic Branch [40]. Two other solutions are Pr(p)=1, Pr(d)=1-Pr(p)=0 and Pr(p)=0, Pr(d)=1-Pr(p)=1 (blue and red). We refer to these solutions as the kinetic branch. It should be noted that $J_{0}=0$ represents the conditions typically used to determine ligand binding to receptor equilibrium constants (binding isotherms) and rate constants (dissociation rate).

The solutions in Figure 2 represent the local maximums of information transmission. The capacity of the system is at a local maximum in the solutions. Two parameters determine the system output, the change in free energy $\beta \left(E_{p}-E_{d}\right)$ and the externally driven flux $J_{0}$. Rapid shifts in these two quantities may initiate a shift from one local maximum to another.

For most pharmaceutical applications, the control parameters are altered by changes in the ligand and ligand conformation. Of particular importance are jumps initiated by a change in ligand or ligand conformation that move the switch from the “on position, Pr(p)→1 to the “off position, Pr(d)=1-Pr(p)→1. One type of jump among solutions is particularly important, jumps in which changes in ligand conformation lead to changes in the switch position. This permits the ligand, which may be a drug, to control the downstream cellular response to the ligand. In this study, we focus on the identification of these jumps in the observations in [55]. This informs the efficacy of a drug candidate.

## Appendix B. Connection Between Experiment and Theory

The experimenters in [55] assumed the existence of a strong correlation between the measured impedance and the downstream response. We explicitly assume the simplest relationship between impedance and response, which is linear

(B.1)
$$Pr\left(d\right)=1-Pr\left(p\right)=\frac{Z\;-\;Z_{DMSO}}{Z_{hPP}\;-\;Z_{DMSO}},$$

where $Z_{hPP}$ is the impedance measured with the endogenous agonist human pancreatic polypeptide (hPP) and $Z_{\text{DMSO}}$ is the impedance for the solvent DMSO (negative control). The Biological Ensemble is symmetric upon interchange of p and d. Therefore, Eq. B.1 with p replaced with d is also a possible connection between experiment and theory. As long as the relationship between Pr(p) and Z is monotonic, the conclusions of this paper should not be affected by the exact form of Eq. B.1. hPP and DMSO were used to define the boundaries of observable impedance in [55]. The connection between impedance Z and probability of the “on” position Pr(d) provides visibility into how flux $J_{0}$ and change in free energy $\beta \left(E_{p}-E_{d}\right)$ change in response to various ligand molecules and conformations of the molecules. Figure 2 provides the map for the relationship between impedance and control parameters $J_{0}$ and change in free energy for each ligand.

## Appendix C. Information Landscape

Figure C.6 presents a conceptual schematic of the information landscape described in Section 3.2, with the two control parameters, $\beta \left(E_{p}-E_{d}\right)$ and $J_{0}$, plotted along the horizontal axes and the information content of the system on the vertical axis. The surface contains peaks and valleys, representing points where the time derivative of the information content is zero. These extrema correspond to the NESS of the system. The specific NESS for the modeled system are mapped in Figure 2. Each ridge in Figure C.6 marks a local information maximum, although not necessarily the global maximum.

In Figure C.7, the global maxima of the Biological Ensemble solutions are shown in black. Within this model, these black trajectories correspond to the highest possible information transmission and thus represent the most probable states of the system. The remaining trajectories represent local maxima, quasistable states that can persist for extended periods, but are less likely to occur naturally. For a system residing in one of these local maxima to transition to the global maximum, it must experience a sufficiently large perturbation to overcome the stability of the local state.

At the critical flux

(C.1)
$$J_{0}=\phi_{c}\approx 0.182$$

The maximum information in the thermodynamic branch and the kinetic branch is equal at free energy $\beta \left(E_{p}-E_{d}\right)$ For flux greater than $\phi_{c}$ two small isolated solution islands appear as seen in Figures C.7D-G. As the flux $J_{0}$ increases further, the regimes eventually merge into a single continuous solution at the maximum information value of $J_{0}=1/2$, as seen in Figure C.7I.

Figures C.7 B-I illustrate the three solutions as the flux $J_{0}$ increases. At

(C.2)
$$J_{0}=\frac{1}{4}$$

the thermodynamic branch reduces to the “off” state Pr(p)=0 leaving only the kinetic branch with finite probability for Pr(p). The thermodynamic branch contains zero entropy and information for $J_{0}>1/4$. Figs. 2G-I illustrate the supercritical behavior of the two solutions of the kinetic branch in this regime.

It is important to note that the optimal maximum-information solution for the NESS is discontinuous. Phase transitions occur within the phase space defined by the control parameters $J_{0}$ and $\beta \left(E_{p}-E_{d}\right)$. This implies that for a given flux $J_{0}$, a minor change in the binding energy can lead to a significant alteration in the switch state - “on” or “off”, particularly when the energy is adjusted near a phase transition. Moreover, the system can be prepared to lie on paths of local maximum of capacity. These states can transition to the global maximum with a sufficient perturbation.

## 9. Glossary

ATP: Adenosine Triphosphate
CHO: Chinese hamster ovary
GPCR: G Protein Coupled Receptor
GTP: Guanosine Triphosphate
NESS: Nonequilibrium Steady State
